# Preclinical evaluation of the ROCK1 inhibitor, GSK269962A, in acute myeloid leukemia

**DOI:** 10.3389/fphar.2022.1064470

**Published:** 2022-12-06

**Authors:** Ting Pan, Sijia Wang, Hao Feng, Jiawen Xu, Miao Zhang, Yao Yao, Kailin Xu, Mingshan Niu

**Affiliations:** ^1^ Blood Diseases Institute, Xuzhou Medical University, Xuzhou, Jiangsu, China; ^2^ Department of Hematology, Affiliated Hospital of Xuzhou Medical University, Xuzhou, Jiangsu, China

**Keywords:** acute myeloid leukemia, targeted therapy, ROCK1, GSK269962A, ERK

## Abstract

Acute myeloid leukemia (AML) is an aggressive hematologic malignancy with high mortality that urgently requires new treatments. ROCK1 plays an essential role in regulating growth and survival in AML cells. In this study, we evaluated GSK269962A, a selective ROCK1 inhibitor, in preclinical models of AML. Compared with solid tumors, GSK269962A selectively inhibited cell growth and clonogenicity of AML cells. Furthermore, GSK269962A arrested AML cells in the G2 phase and induced apoptosis by regulating multiple cell cycle- and apoptosis-associated proteins. Strikingly, GSK269962A could eliminate leukemia cells from bone marrow, liver, and spleen in an animal model of AML and significantly prolong mouse survival. Mechanistically, GSK269962A could inhibit the growth of AML by blocking ROCK1/c-Raf/ERK signaling pathway. Notably, a correlation was found between the expression levels of ROCK1 protein and the sensitivity of GSK269962A in AML. These data highlight the potential role of ROCK1 as an attractive target for treating AML, as well as the potential of GSK269962A for use in clinical trials of AML.

## Introduction

Acute myeloid leukemia (AML) is caused by malignant clonal proliferation of myeloid hematopoietic stem/progenitor cells and is the most common form of acute leukemia in adults (Carter et al., 2020; Yamashita et al., 2020). The 5-year overall survival rate for adult AML patients <60 years old with standard chemotherapy is only about 40%, and most patients eventually relapse and die. As many elderly AML patients aged ≥60 years cannot tolerate standard doses of chemotherapy, the median survival time is only 5–10 months (LeBlanc and Erba, 2019; Herold et al., 2020). In addition, most patients have no genetic mutations that can be directly targeted (Gebru and Wang, 2020). Therefore, it is imperative to identify new therapeutic targets for AML.

Rho-associated kinases (ROCKs) are the downstream effectors of RhoA small GTPase (Deng et al., 2019). ROCK1 regulates cell functions, including migration, apoptosis, survival, and proliferation (Zhang et al., 2019; Landry et al., 2020; Lu et al., 2020). Although the key roles of ROCK1 in solid tumor metabolism have been well elucidated, its role in leukemogenesis is only partly explained (Mali et al., 2014). In the KIT (D814V)-induced myeloid leukemia mouse model, knockout of ROCK1 increased survival in all mice, while all wild-type control mice died throughout the experiment (Mali et al., 2011). Recently, ROCK1 was identified as a crucial gene for leukemic bulk and stem/progenitor cells using large-scale RNAi screens in primary human AML cells (Wermke et al., 2015). DJ4, a novel ROCK inhibitor, was demonstrated to suppress ROCK/MYPT1/MLC2 pathway and result in cell death of AML cells (Golla et al., 2021). As these findings suggested that ROCK1 could be a potential therapeutic target for AML, evaluating the anti-leukemia effect of ROCK1 inhibitor in preclinical models might be of potential value.

Given the important roles of ROCK1 in diseases, an increasing number of inhibitors have been developed, most of which inactivate ROCK1 in a competitive manner with ATP (Shahbazi et al., 2020). Fasudil is the first ROCK1-targeted drug approved for clinical use in the treatment of cerebral vasospasm. It has been reported that fasudil has minimal and acceptable side effects (Wei et al., 2016). However, fasudil has low potency in inhibiting ROCK1, and its activity of inhibiting tumor cell growth *in vitro* is at the micromolar level, which is too high to achieve *in vivo* effect. GSK269962A can potently inhibit ROCK activity, with IC_50_ values of 1.6 and 4 nM for recombinant ROCK1 and ROCK2, respectively. GSK269962A also has an improved kinase selectivity profile with at least >30-fold selectivity against the panel of protein kinase tested (Doe et al., 2007). Thus, its potential anti-leukemia effect in an AML model is worth evaluating. In this study, we evaluated the preclinical efficacy and biological activity of GSK269962A in AML. Furthermore, we analyzed the mechanisms through which GSK269962A inhibits the growth of AML.

## Materials and methods

### Cell cultures

Human AML cell lines MV4-11, OCI-AML3, NOMO-1, MOLM-13, THP-1, Kasumi-1, and KG-1 cells were cultured in RIPM-1640 medium containing 10% FBS in a humidified incubator containing 5% CO_2_ at 37°C. Primary normal hematopoietic cells were obtained from healthy donors who donated peripheral blood stem cells for allogeneic hematopoietic stem cell transplantation following mobilization by G-CSF. The primary cells were cultured in Myelo-Cult™ H5100 media (Stem Cell Technologies) supplemented with rh-FLT3-L (50 ng/ml), rh-SCF (50 ng/ml), rh-IL6 (20 ng/ml), and rh-IL-3 (20 ng/ml). The acquisition of primary samples was approved by Ethics Committee of the Affiliated Hospital of Xuzhou Medical University.

### Reagents

Primary antibodies against Cyclin A2 (4656S), Cyclin E1 (20808S), Cyclin B1 (12231S), Cdc25C (4688S), Cdc2 (28439S), CDK6 (13331S), p53 (2524SS), p-p53 (9286S), PARP (9532S), Mcl-1 (94296S), Survivin (2808S), Bcl-xL (2764S), ROCK1 (4035S), p-Akt (4058S), Akt (4685S), p-c-Raf (9427S), p-MEK1/2 (9154S), MEK1/2 (8727S), p-ERK (9106S), ERK (4695S), *β*-Actin (4970S) and Vinculin (13901S) were purchased from Cell Signaling Technology (MA, United States). ROCK1 inhibitor GSK269962A was obtained from TargetMol Chemicals Inc. (MA, United States).

### Cell viability assay

AML cells were seeded in 96-well plates at 10,000 cells per well, and incubated with different concentrations of GSK269962A. After 72 h, 10 μl of CCK-8 solution was added to each well and incubated at 37°C for 2 h. The OD value at 450 nm was measured with a microplate reader. Background reading of the media was subtracted from each well for result standardization.

### EdU incorporation assay

Cell proliferative capacity was detected using the kFlour647 ClickiT EdU Flow Detection Kit (KeyGEN BioTECH) (Zhang et al., 2021). AML cells were seeded in 6-well plates and cultured for 48 h with or without GSK269962A. Cells were then incubated with 20 μM EdU for 4 h after drug exposure, after which they were washed three times and then treated with fixation and permeabilization buffer (BD Biosciences) for 30 min. Next, cells were washed and incubated with 200 μl of iClick reaction solution for 30 min. The EdU-positive rate was measured by flow cytometry and was analyzed by the FlowJo software.

### Colony-formation assay

AML cell lines (300 cells/dish) or primary cells (5,000 cells/dish) were seeded into 35 mm culture dishes with MethoCult™ H4435 Enriched medium-plus recombinant cytokines (STEMCELL TECHNOLOGIES). Cells were cultured with or without GSK269962A for 12 days in a 5% CO_2_ humidified atmosphere at 37°C. The size of colonies was assessed by a microscope, and the number of colonies was counted. The data presented include three replicates in each treatment.

### Cell cycle assay

MV4-11 and OCI-AML3 cells were seeded in 6-well plates, treated with the indicated concentration of GSK269962A, and cultured for 12 h. Then, cells were collected and fixed with 70% pre-chilled ethanol overnight, washed with PBS, and stained with 200 μl PI/RNase Staining Buffer for 30 min in the dark. After being washed three times with PBS, cycle distributions of cells were analyzed with ModFit LT 5.0 software by flow cytometry.

### Cell apoptosis assay

Apoptosis was detected using Fluorescein isothiocyanate (FITC) annexin V Apoptosis Detection Kit I (BD Biosciences) according to the manufacturer’s instructions. Briefly, AML cells (10^6^ cells/well) were seeded in 6-well plates and cultured for 48 h with or without GSK269962A, after which they were collected, washed twice with pre-cooled PBS, and resuspended in 200 μl of Binding buffer. Next, 5 μl Annexin V-FITC and 5 μl PI were added to cells, and cells were incubated on ice for 20 min in the dark. Cell apoptosis was detected and analyzed by flow cytometry.

### Caspase-Glo 3/7 activity assay

AML cells were seeded into 96-well plates and treated with GSK269962A for 24 h. Caspase-Glo 3/7 enzymatic activities were measured according to the manufacturer’s protocol (Promega). Then, 100 μl of Caspase-Glo 3/7 reagent was added to the cells and was mixed well. After 30 min, 200 μl of the solution was transferred into a white-walled multiwell luminometer plate. The GloMax Luminometer was used to determine the luminescence signal value of samples in each group.

### Western blot

Cells were seeded into 6-well plates and treated with indicated concentrations of GSK269962A, after which they were harvested, and total protein was extracted with cell lysate (Cell Signaling Technology). Proteins were separated by SDS-Page electrophoresis and transferred to PVDF membranes. Membranes were blocked with 5% nonfat dry milk for 1 h and incubated overnight at 4°C with the corresponding primary antibodies, after which they were washed and incubated with secondary antibodies for 1 h. ECL substrate (Bio-Rad) and chemiluminescence imager (ImageQuant LAS 4000) were used for detection.

### *In vivo* studies

The female NOD-SCID/IL2Rgnull (NPG) mice (Vitalstar Biotechnology, China) were used to establish the AML model. All animal experimental protocols were approved by the ethics committee of the Xuzhou Medical University. The MV4-11 cells (1 × 10^6^) were intravenously administered to 6–8-week-old NOD-SCID/IL2Rgnull mice. After 3 days of administration, these mice were randomly divided into three groups: control group, 5 mg/kg group, and 10 mg/kg group, which were administered by intraperitoneal injection for 5 days per week. The test GSK269962A was dissolved in 20% PEG300/0.25% Tween-80/79.75% water for administration. After 4 weeks of administration, three animals in each group were randomly selected and sacrificed for pathological analysis. The remaining mice were used for survival analysis.

The toxicity of GSK269962A was assessed by treating C57BL/6 mice with vehicle (*n* = 5) or GSK269962A (10 mg/kg, *n* = 5) for 10 days. Blood samples were obtained from the animals and complete blood count (CBC) was analyzed using an automated hematology analyzer. The vital organs were collected for histo-pathological analysis. The organs (kidney, liver, lung, and spleen) were fixed in formalin and assessed tissue morphology with hematoxylin and eosin (H&E) staining.

### Hematoxylin-eosin staining and immunohistochemistry

Tissues were fixed in 4% paraformaldehyde overnight (Wang et al., 2020). After dehydration, the tissue was embedded in paraffin and then cut into sections. H&E staining was performed using deparaffinized sections. For immunohistochemistry, the sections were blocked and incubated with primary antibody CD45 (Cell Signaling Technology).

### Statistical analysis

The data are expressed as the mean ± SD. The experimental results were statistically analyzed using GraphPad Prism 6.0. Comparisons of the mean values between the control and treated groups were performed using Student’s *t*-test. The Kaplan-Meier method was used for the survival analysis of the mice. *p* values < 0.05 was considered statistically significant.

## Results

### ROCK1 inhibitor selectively suppresses the proliferation of AML cells

**FIGURE 1:**
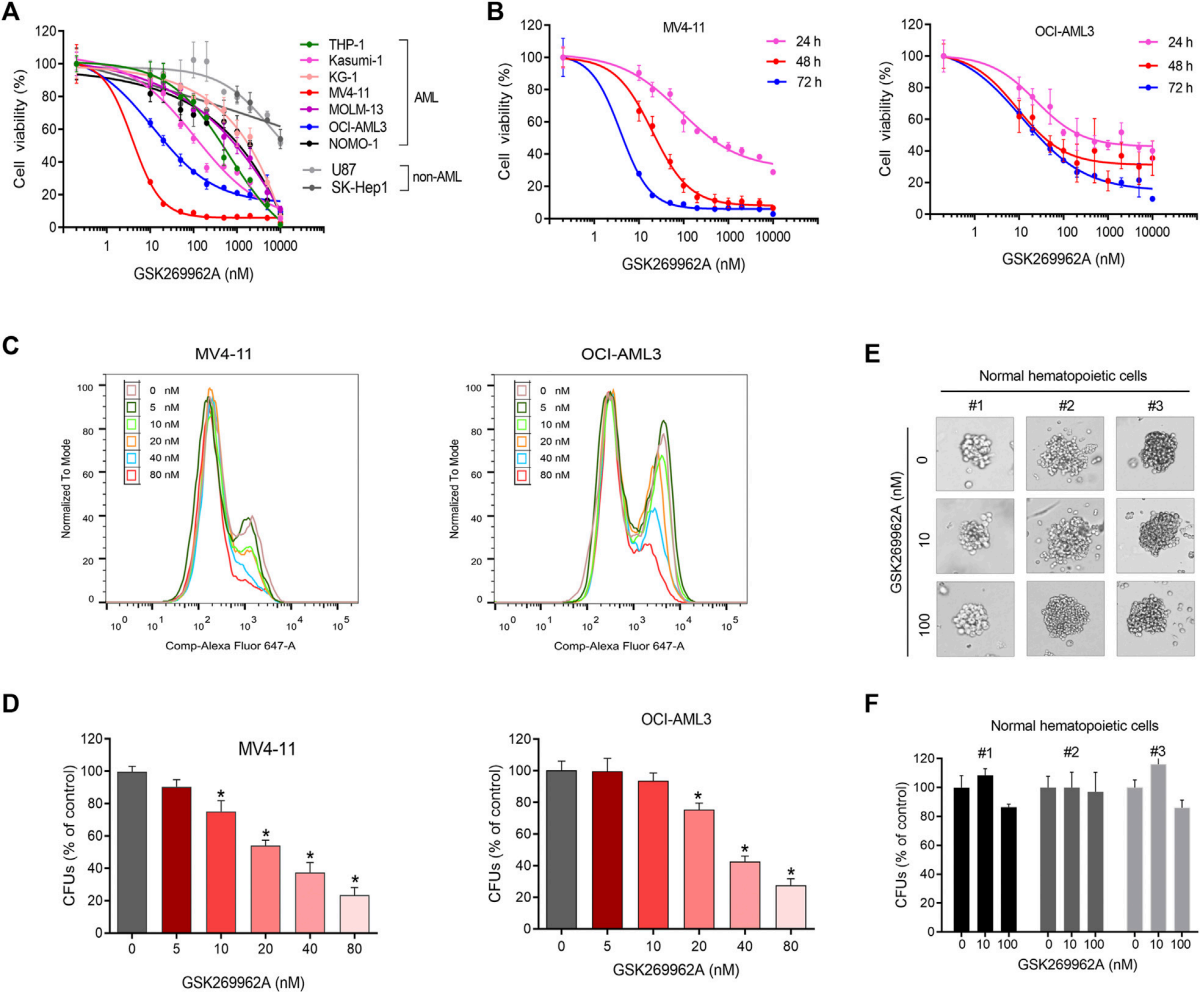
GSK269962A selectively inhibits AML cell growth. **(A)** AML and non-AML cell lines were treated with indicated concentrations of GSK269962A for 72 h. Cell viability measured by a CCK-8 assay. **(B)** MV4-11 and OCI-AML3 cells were treated with GSK269962A for indicated time. Cell viability measured by a CCK-8 assay. **(C)** EdU assay was used to detect the inhibitory effect of GSK269962A on MV4-11 and OCI-AML3 cell proliferation. **(D)** MV4-11 and OCI-AML3 cells were cultured in a methylcellulose medium with or without GSK269962A for 12 days. The number of colonies were analyzed. **(E,F)** A colony-formation assay was performed to evaluate the effect of GSK269962A on the clonogenic ability of primary normal hematopoietic cells.

CCK-8 was performed to evaluate the effect of ROCK1 inhibitor GSK269962A on the growth of seven AML cell lines and three non-AML cell lines. GSK269962A selectively inhibited the growth of AML cells, while non-AML cells were resistant to ROCK1 inhibition (Figures 1A,B). The inhibitory activity (IC_50_) of GSK269962A on AML cells ranged from 0.61 to 1,337 nM (Supplementary Table S1), indicating that the sensitivity of different cell lines to ROCK1 inhibition varied greatly. MV4-11 and OCI-AML3 with higher sensitivity were selected for follow-up studies.

To determine the effect of GSK269962A on AML proliferation, an EdU assay was used to detect the proliferation ability of AML cells. GSK269962A significantly decreased the ratio of EdU-positive cells in MV4-11 and OCI-AML3 cells compared with the control group in a dose-dependent manner (Figure 1C). These data suggested that GSK269962A could selectively inhibit the proliferation of AML cells.

### GSK269962A suppresses the colony-formation of AML cells

The colony formation assay was conducted to evaluate the long-term inhibitory effect of GSK269962A on the proliferation of AML and normal hematopoietic cells. GSK269962A significantly reduced the size of AML cell colonies compared to the control group. TAK-243 also significantly reduced the number of MV4-11 and OCI-AML3 colonies formed in a dose-dependent manner (Figure 1D; Supplementary Figure S1). However, GSK269962A had no significant effect on the size and number of primary normal hematopoietic cell colonies (Figures 1E,F). These results demonstrated that GSK269962A can abolish the clonogenic growth of AML cells.

### GSK269962A induces G2 arrest and modulates the expression of cell cycle regulators

**FIGURE 2:**
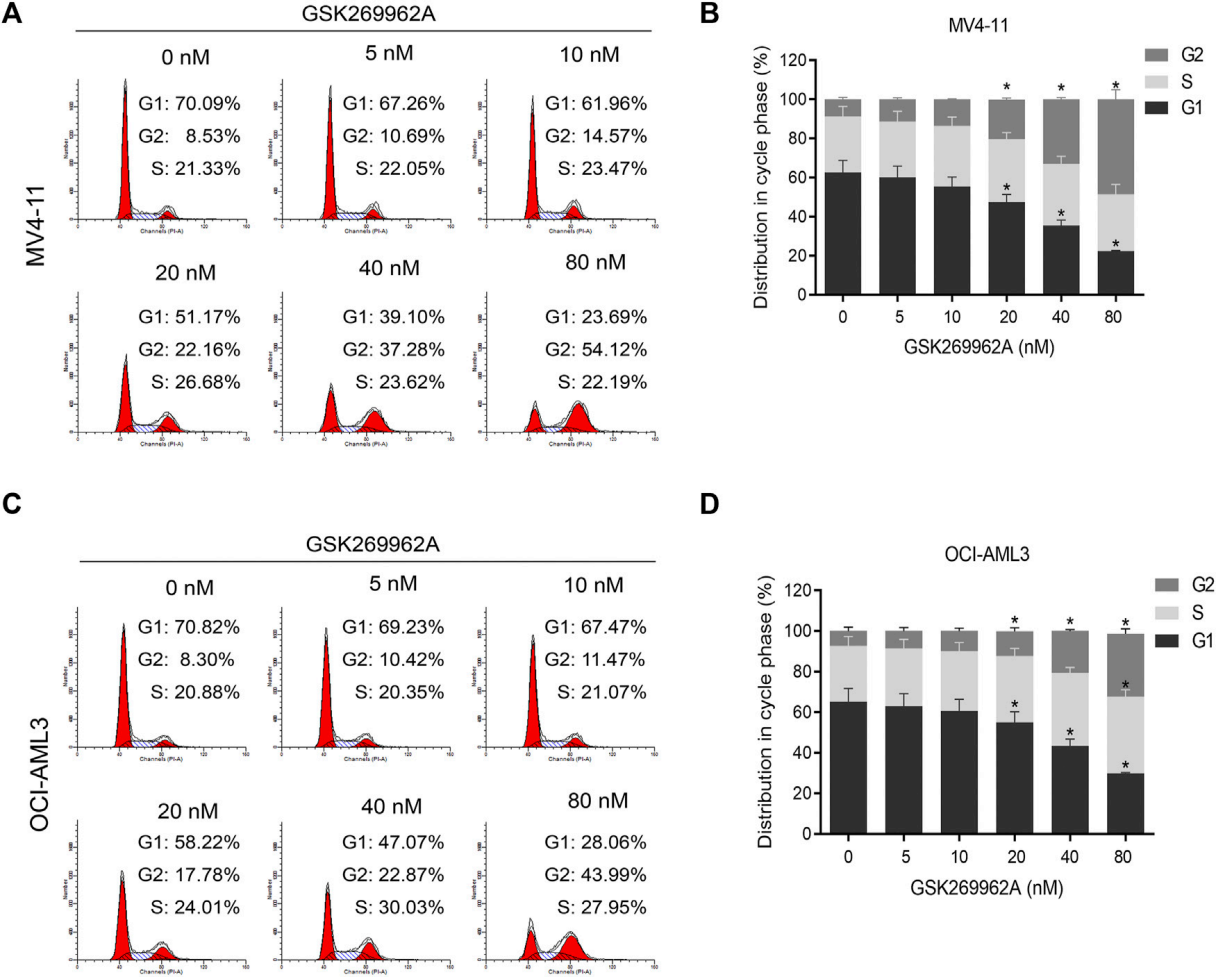
ROCK1 inhibitor GSK269962A induces G2 phase arrest of AML cells. **(A,C)** MV4-11 and OCI-AML3 cells were treated with indicated concentrations of GSK269962A. The cells were stained with propidium iodide and evaluated using a flow cytometer. **(B,D)** Quantitative analysis of cell-cycle-phase distributions in each group.

In order to determine whether GSK269962A-induced reduction in cell proliferation by cell cycle arrest, we performed flow cytometry. In MV4-11 and OCI-AML3 cells, GSK269962A induced a significant increase in the number of cells in the G2 phase, with a corresponding decrease in the number of cells in the G1 phase (Figures 2A–D). Also, the number of cells in the G2 phase in the control group was only about 8%, while the number of cells in the G2 phase increased to about 50% after treatment with 80 nM GSK269962A.

Next, we examined the expression levels of related regulatory proteins during the cell cycle. As shown in Figure 4A, GSK269962A significantly decreased the protein expression levels of phosphatase Cdc25C, Cyclin-Dependent Kinase CDK6, Cyclin family Cyclin A2, Cyclin B1, and Cyclin E1. These proteins have important roles in the regulation of G2/M in AML cells. These data indicated that GSK269962A could inhibit AML cell proliferation by regulating multiple cell cycle-related proteins and inducing G2 arrest.

### ROCK1 inhibition induces apoptosis of AML cells

**FIGURE 3:**
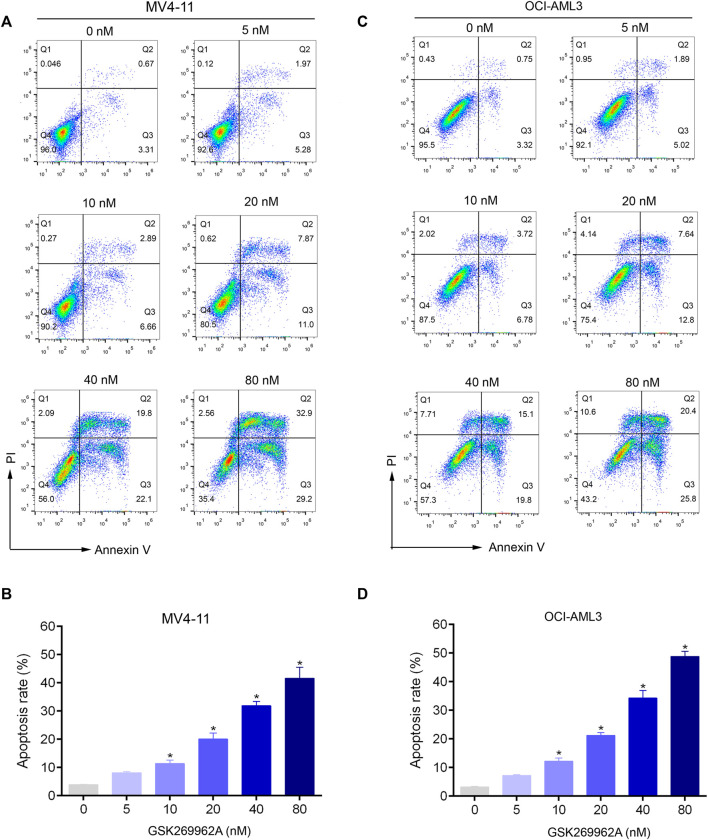
GSK269962A induces apoptosis of AML cells in a dose-dependent manner. **(A,C)** MV4-11 and OCI-AML3 cells were treated by increasing concentrations of GSK269962A. The cells were stained with Annexin V/PI and analyzed using a flow cytometer. **(B,D)** Quantification of annexin-V–positive cells in each group.

To evaluate the effect of GSK269962A on AML cell apoptosis, Annexin V/PI apoptosis assay was performed. Compared with the control group, GSK269962A significantly increased the percentage of apoptotic cells in a dose-dependent manner. Apoptotic ratios of MV4-11 and OCI-AML3 cells increased to more than 40% by treatment of 80 nM GSK269962A (Figures 3A–D).

**FIGURE 4:**
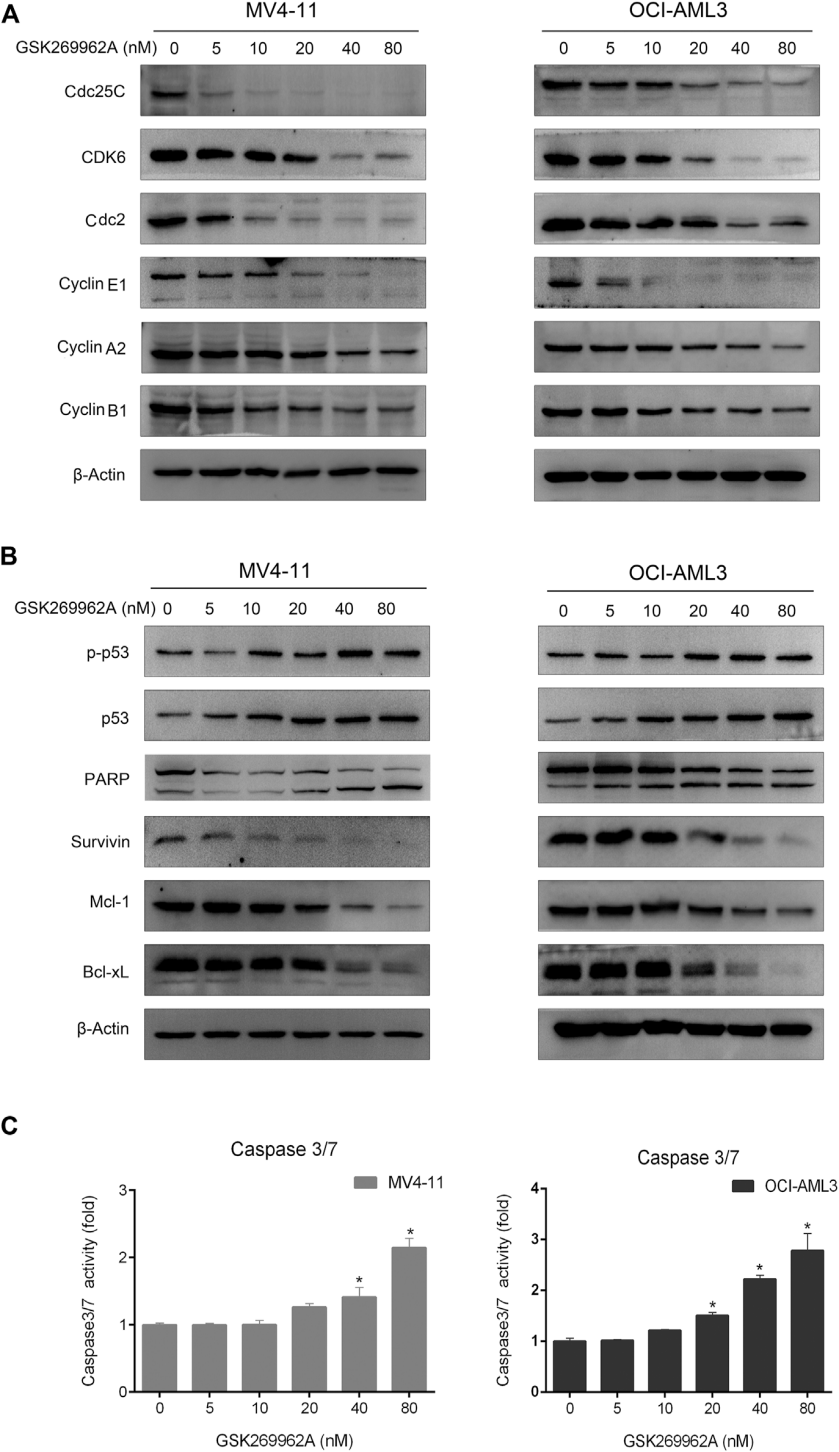
GSK269962A modulates the expression of cell death regulators. **(A,B)** MV4-11 and OCI-AML3 cells were treated with indicated concentrations of GSK269962A. The protein extracts were examined using Western blot analysis with the indicated antibodies. **(C)** Caspase-3/7 activity was assessed using a luminescent assay.

Next, we examined the effect of GSK269962A on the expression and activity of apoptosis-related regulatory proteins. As shown in Figure 4B, both the expression and phosphorylation level of the pro-apoptotic protein p53 increased with the increasing concentration of GSK269962A, and the PARP was cleaved into an active spliceosome. Besides, the expression levels of anti-apoptotic proteins including Survivin, Mcl-1, and Bcl-xL significantly decreased in MV4-11 and OCI-AML3 cells. The enzymatic activity of Caspase-3/7 also increased with the increasing concentration of GSK269962A (Figure 4C). These data suggested that GSK269962A strongly induces the apoptosis of AML cells by regulating multiple apoptosis-associated proteins.

### GSK269962A reduces the leukemic burden in a mouse xenograft model

**FIGURE 5:**
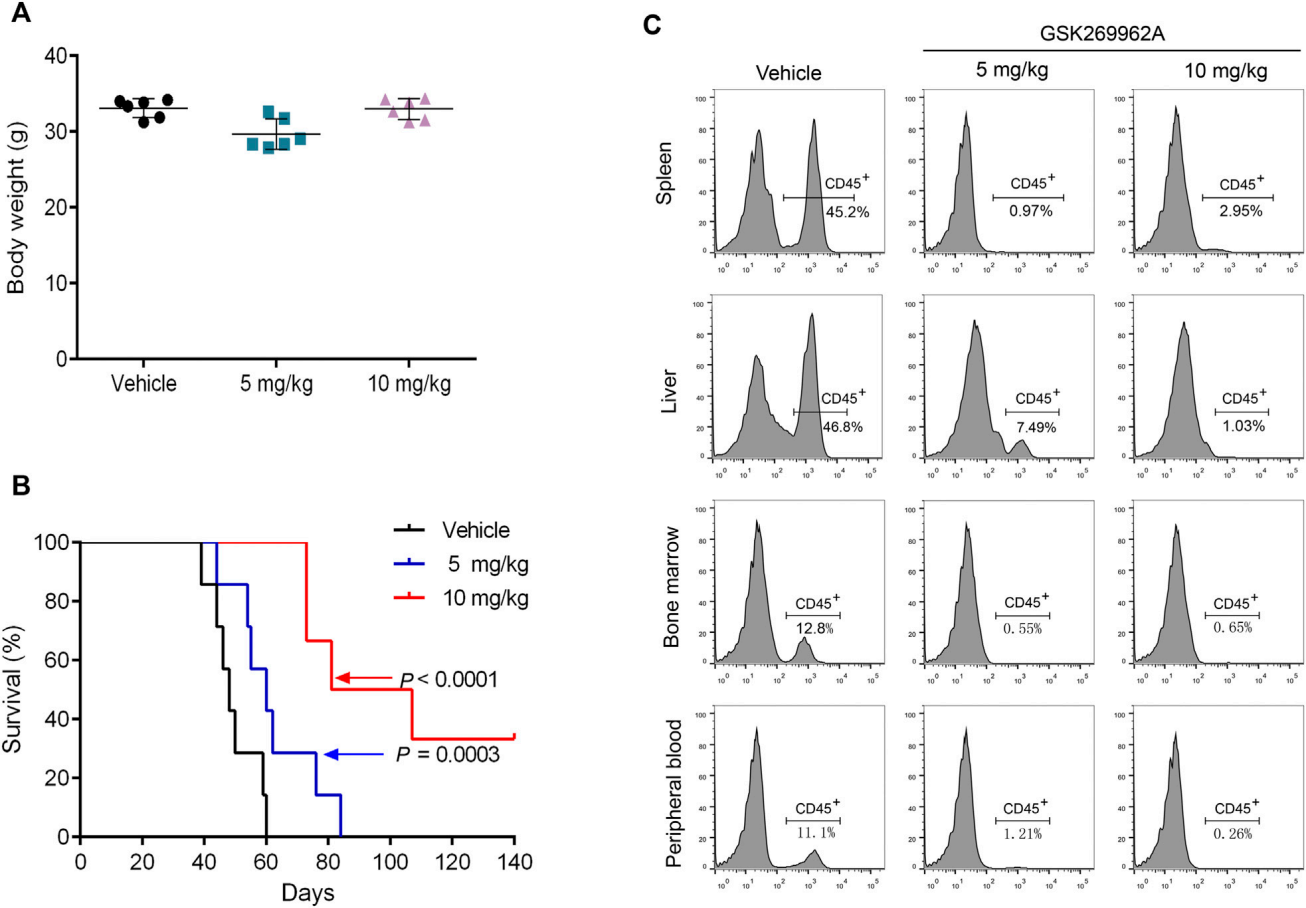
GSK269962A reduces the leukemia progression *in vivo*. MV4-11 cells were injected into NPG mice *via* tail vein injections. Mice were treated with GSK269962A (5 or 10 mg/kg) or vehicle control by intraperitoneally injection 5 days a week for 4 weeks. **(A)** The mice were weighed at the end of GSK269962A administration. **(B)** The survival of mice was analyzed by Kaplan-Meier survival curves. **(C)** Flow cytometry was used to determine the proportion of human CD45 positive cells in peripheral blood, bone marrow, spleen and liver.

To assess the efficacy of GSK269962A *in vivo*, we used a mouse xenograft model of AML. The AML model was established by tail vein injection of MV4-11 cells into NOD-SCID/IL2Rgnull mice. After 4 weeks of administration, the body weight of the mice did not significantly change, thus indicating that the dose and schedule of the drug were well-tolerated (Figure 5A). Furthermore, GSK269962A significantly prolonged the survival of mice with a median survival time of 49 days in the control group, 61 days in the 5 mg/kg group, and 94 days in the 10 mg/kg group (Figure 5B). Notably, some mice in the 10 mg/kg group achieved complete response and were still alive more than 100 days after suspension of GSK269962A. Next, we measured the ratio of hCD45-positive AML cells in each organ by flow cytometry. As shown in Figure 5C, GSK269962A with 10 mg/kg almost completely eliminated hCD45-positive cells in PB, BM, spleen, and liver.

**FIGURE 6:**
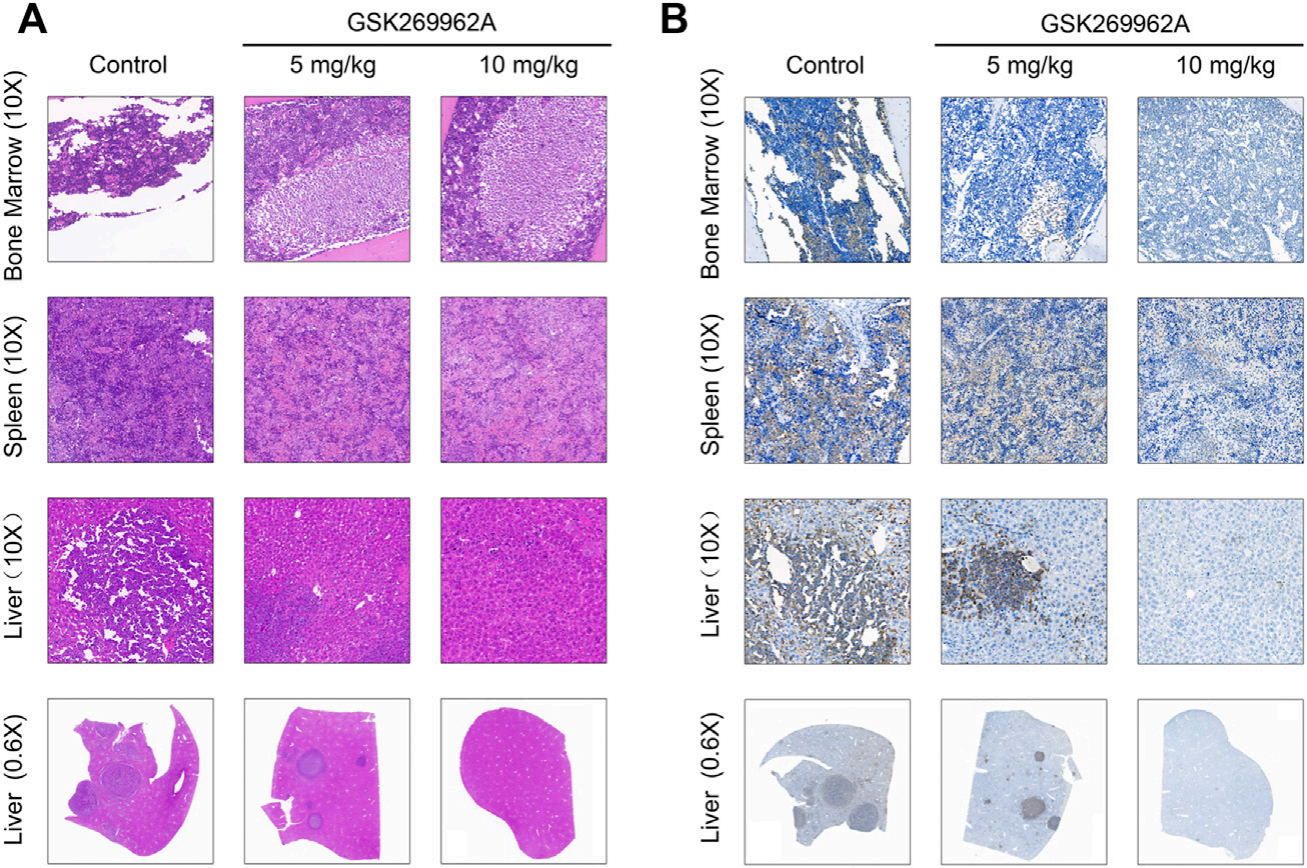
The pathological morphologies of mouse tissues. **(A)** H&E staining for the indicated tissues in animals treated with control or GSK269962A. **(B)** IHC analysis of the human CD45 positive cells in the bone marrow, liver, and spleen of mice.

Moreover, we performed a pathological analysis of mouse BM, spleen, and liver by H&E and IHC. As shown in Figure 6A, the infiltration of AML cells could be found in each organ of mice in the control group after H&E staining. The AML cells in the bone marrow were severely infiltrated, and a large number of normal cells disappeared, leaving a cavity with focal necrosis. However, GSK269962A significantly reduced the infiltration of leukemia cells in various organs. Consistently, IHC also showed that GSK269962A significantly reduced the ratio of hCD45-positive cells in BM, spleen, and liver compared with the control group (Figure 6B). Notably, there were multiple large hCD45-positive leukemia foci in the liver of mice from the control group, whereas the enrichment of leukemic cells in mice from the 10 mg/kg administration group was almost completely eliminated. Taken together, these data showed that pharmacological inhibition of ROCK1 by GSK269962A strongly suppresses the proliferation of the AML cells *in vivo* and prolongs the survival of the leukemic mice.

To examine potential cytotoxic side-effects of GSK269962A on normal tissues, C57BL/6 mice were treated with vehicle or GSK269962A, and whole blood samples as well as vital organs were collected for hematology and histopathological analyses, respectively. These data from the standard hematological evaluation did not reveal any significant difference between the control and GSK269962A treated groups (Supplementary Table S2). Histopathological evaluation of the H&E stained slides of the organs (kidney, liver, lung, and spleen) did not reveal any significant difference between the control and GSK269962A treated groups (Supplementary Figure S2). Together, these results indicate that treatment with GSK269962A without any overt signs of harmful effects to their system.

### GSK269962A blocks ROCK1/c-Raf/ERK pathway in AML cells

**FIGURE 7:**
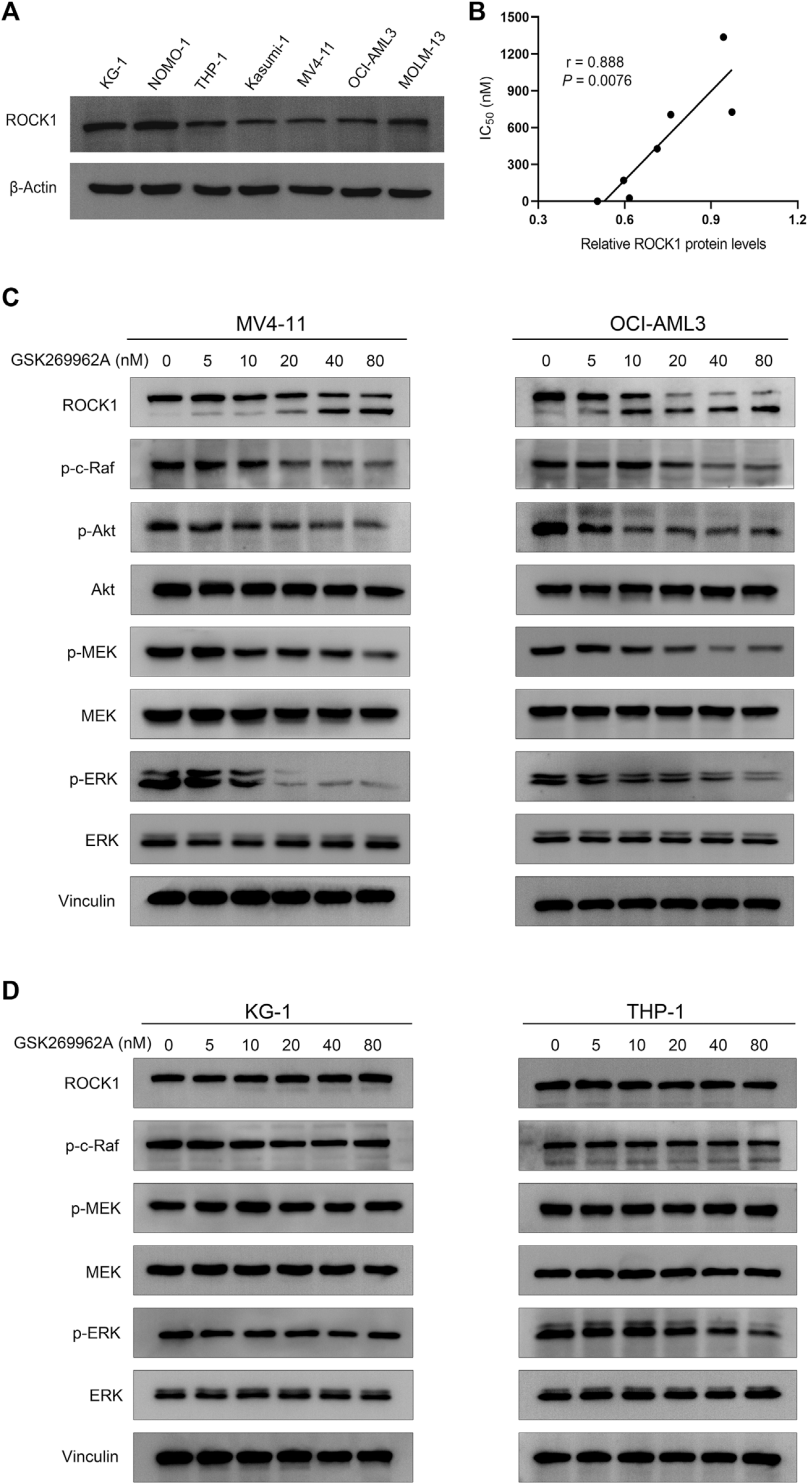
GSK269962A suppresses ROCK1/c-Raf/ERK pathway in AML cells. **(A)** The expression level of ROCK1 was examined by Western blotting in AML cell lines. **(B)** Correlation analysis of relative ROCK1 protein levels and IC_50_ values of GSK269962A in AML cell lines. **(C,D)** MV4-11, OCI-AML3, KG-1, and THP-1 cells were treated with GSK269962A and processed for Western blotting with the indicated antibodies.

Considering ROCK1 is an effector of Rho GTPases (Svensmark and Brakebusch, 2019), we first examined the effect of GSK269962A on the phosphorylation levels of Akt and ERK that may be cascaded by Rho Kinase. We found that GSK269962A reduced ERK phosphorylation more strongly than Akt phosphorylation, thus indicating that GSK269962A mainly regulated ERK signaling pathway in AML cells. GSK269962A also inhibited the phosphorylation levels of c-Raf and MEK in this cascade without affecting total protein expression levels (Figure 7C). Notably, we found that GSK269962A contributed to the cleavage of ROCK1, as with the increase of GSK269962A concentration, the expression level of the full-length form of ROCK1 gradually decreased. Moreover, the expression of spliceosome of ROCK1 gradually increased. These data suggest that GSK269962A can inhibit ROCK1/c-Raf/ERK signaling pathway in AML cells.

### The expression levels of ROCK1 are correlated with GSK269962A sensitivity in AML cells

Since the sensitivity of GSK269962A to different AML cells was different, we analyzed the correlation between expression level and sensitivity of ROCK1. As shown in Figure 7A, the expression level of ROCK1 varied in different AML cell lines. Interestingly, there was a significant correlation between the expression level of ROCK1 and GSK269962A (Figure 7B). Next, we found that the same concentration of GSK269962A failed to induce ROCK1 degradation in insensitive KG-1 and THP-1 cells. GSK269962A also had no significant effect on the phosphorylation levels of c-Raf, MEK, and ERK (Figure 7D). To sum up, these results suggested that the expression levels of ROCK1 were correlated with GSK269962A sensitivity in AML cells.

## Discussion

AML is an aggressive hematologic malignancy with high mortality that urges the need for the exploration of novel therapeutic strategies (Winer and Stone, 2019). In this study, we evaluated the anti-leukemia effect of GSK269962A in a preclinical model of AML. GSK269962A effectively inhibited the growth of AML cells without significant inhibition of other tumor cells. Notably, GSK269962A could effectively eliminate the infiltration and enrichment of leukemia cells in various organs in AML animal models and significantly prolong mice survival. Mechanistically, GSK269962A inhibited ROCK1/c-Raf/ERK signaling pathway in AML cells.

Cell-cycle dysregulation and apoptosis inhibition of AML cells conferred them the ability of clonal proliferation (Jammal et al., 2020). We found that low nanomolar levels of GSK269962A could arrest AML cells in the G2 phase and induce apoptosis. Cdc25C is a cyclin of the specific phosphatase, which can activate the Cyclin B1-Cdc2 complex. The entry of cancer cells into mitosis is regulated by activation of Cyclin B1-Cdc2 at the G2/M transition (Liu et al., 2020). Cyclin A2 is an essential regulator of the cell division cycle and often found highly expressed in human cancers (Loukil et al., 2015). The overcoming G1/S checkpoint to commence DNA replication requires Cyclin E1, which can also activate Cdc2 (Caldon and Musgrove, 2010). CDK6 can connect cell-cycle progression to angiogenesis and have a central role in hematopoietic malignancies (Kollmann et al., 2013). We found that GSK269962A treatment significantly reduced the expression levels of these cell cycle regulatory proteins. Thus, the anti-proliferative effect of GSK269962A in AML cells may be associated with cell cycle arrest.

The tumor suppressor p53 has a key role in apoptosis and senescence (Hafner et al., 2019; Duffy et al., 2022). Dysfunction of p53 is a frequent occurrence in AML (Barbosa et al., 2019). Mcl-1 and Bcl-xL can interact with and antagonize pro-apoptotic proteins, and inhibit cell apoptosis (Lee et al., 2019). Survivin is an anti-apoptotic protein that binds and inhibits Caspase-3/7 (Shin et al., 2001). PARP is one of the main cleavage targets of caspase-3. The cleavage of PARP facilitates cellular disassembly and serves as a marker of cells undergoing apoptosis (Rouleau et al., 2010). GSK269962A induced the expression level and phosphorylation of p53 to increase at the same time. GSK269962A treatment also decreased the expression of Survivin, Bcl-xL, and Bcl-xL, and induced the cleavage of PARP in AML cells. Taken together, these results suggest that GSK269962A induces apoptosis of AML cells may *via* the mitochondrial-dependent pathway.

Strikingly, GSK269962A could eliminate leukemia cells from bone marrow, liver, and spleen in an animal model of AML, achieving a durable effect. Some GSK269962A-treated mice remained disease-free by the statistical cutoff, surviving at least 140 days. It has also been reported that knockout of ROCK1 enabled the mice to survive throughout the entire duration of the experiment in a KIT (D814V)-induced myeloid leukemia mouse model (Mali et al., 2011). Taken together, these results suggest that ROCK1 is an attractive potential therapeutic target for AML, and GSK269962A can effectively inhibit leukemia proliferation *in vitro* and *in vivo*.

The important functions and mechanisms underlying ROCK1 in solid tumor metabolism have been well elucidated (Rath and Olson, 2012); however, its mechanism in AML is still poorly understood. In their study, Wermke et al. (2015) identified ROCK1 as a crucial gene for leukemic bulk and stem/progenitor cells but did not clarify the signaling pathway regulated in AML. We found that GSK269962A could inhibit the phosphorylation levels of c-Raf, MEK, and ERK kinases in AML cells. These three kinases are important components of the MAPK signaling cascade, contributing to the growth and survival of AML cells (Zhang et al., 2022). ERK is the main downstream effector in MAPK signaling (Cao et al., 2019), while ERK-targeted drugs can also inhibit the malignant proliferation of AML cells (Weisberg et al., 2020). GSK269962A also inhibits MSK1 kinase activity with an IC_50_ value of 49 nM (Doe et al., 2007). MSK1 activation was observed in FLT3-ITD carrying AML cell lines, but not in FLT3-ITD-negative cells (Odgerel et al., 2010). Our results showed that GSK269962A is sensitive to OCI-AML3 cells (FLT3-ITD negative), but not to MOLM13 cells (FLT3-ITD positive). Thus, MSK1 may not be the primary target for the action of GSK269962A in AML. Taken together, these results suggest that GSK269962A may inhibit AML cell growth by regulating ROCK1/c-Raf/ERK signaling pathway.

Most of the targeted inhibitors of ROCK1 disrupt kinase activity through ATP competition, as well as GSK269962A with very strong kinase inhibitory activity (Cantoni et al., 2019; de Sousa et al., 2020). Interestingly, we found that GSK269962A treatment can induce cleavage of ROCK1 protein in AML cells. It has been reported that ROCK1 is a direct cleavage substrate of activated Caspase-3 (Chang et al., 2006). This may be due to the inhibition of ROCK1 activity by GSK269962A, activating self-protection or feedback mechanism of AML cells, leading to the cleavage activation of ROCK1 protein. Compared with solid tumors, GSK269962A selectively inhibited cell growth and clonogenicity of AML cells, which suggests that ROCK1 may have different functions in solid tumors and AML. As previously reported in many studies, ROCK1 mainly regulates tumor metabolism in solid tumor cells whilst having an essential role in the growth and survival of AML cells (Mali et al., 2014; Landry et al., 2020). Notably, AML sensitivity to GSK269962A is also highly variable. We found a correlation between the expression level of ROCK1 and the sensitivity of GSK269962A in AML, which can provide molecular markers for the precise therapy of AML patients by targeting ROCK1.

In summary, GSK269962A exhibited preferential activity toward leukemic versus normal hematopoietic cells. In a preclinical animal model of AML, GSK269962A showed a very strong anti-leukemia effect and significantly prolonged mouse survival. The present study provides a strong basis for further clinical trials of GSK269962A in AML. Moreover, the expression level of ROCK1 can be used as a biomarker for the precision therapy of GSK269962A.

## Data Availability

The original contributions presented in the study are included in the article/Supplementary Material, further inquiries can be directed to the corresponding authors.
