# Supplementary material for: Preclinical evaluation of the ROCK1 inhibitor, GSK269962A, in acute myeloid leukemia
Source: Front Pharmacol. 2022 Dec 6;13:1064470. doi: 10.3389/fphar.2022.1064470 (PMC9763303; doi:10.3389/fphar.2022.1064470)
Supplement: Supplementary file 1 [file Image1.PDF]

Supplementary Material

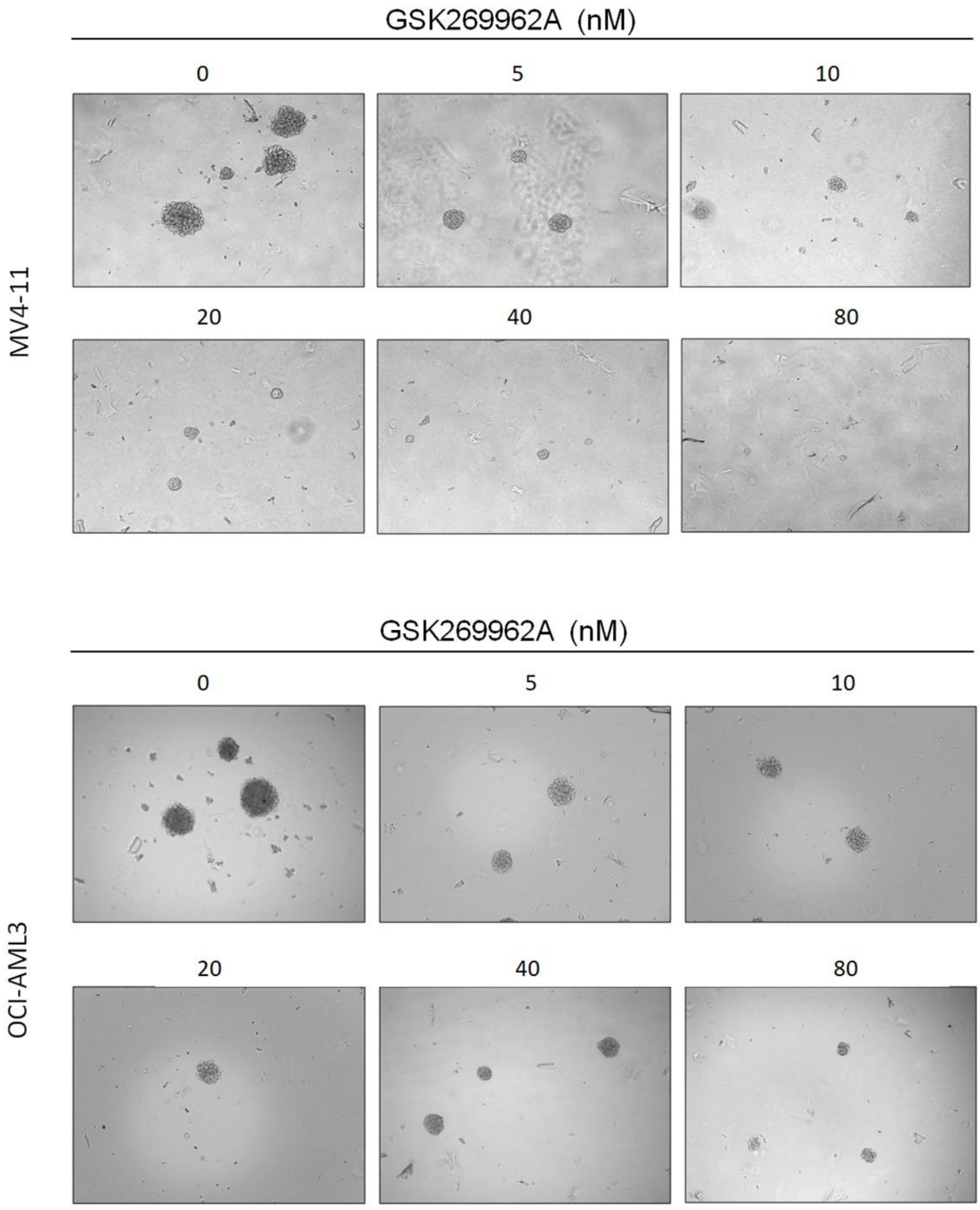

Figure S1. MV4-11 and OCI-AML3 cells were cultured in a methylcellulose medium with or without GSK269962A for 12 days. Microscopic images (20×) of the GSK269962A -mediated effect on clonogenicity of AML cell lines.

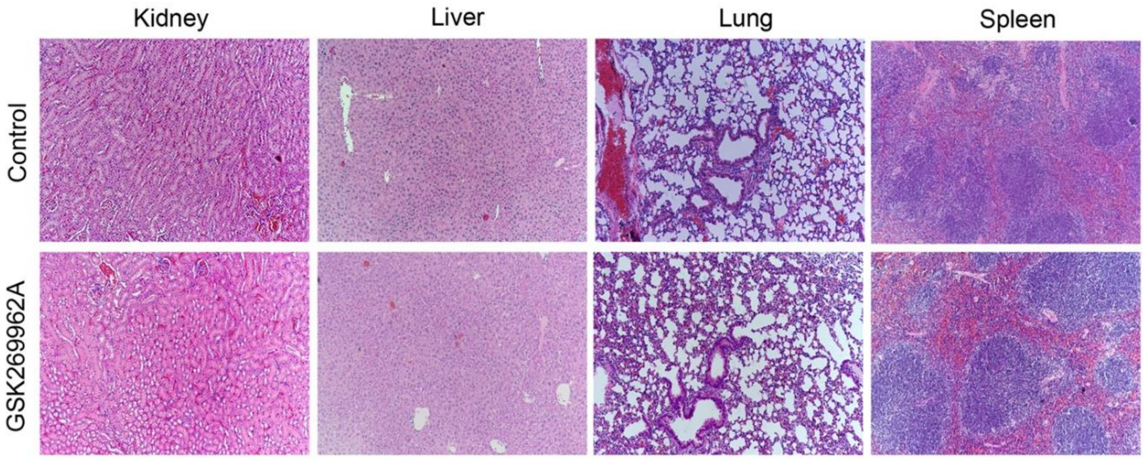

Figure S2. Histological morphology of hematoxylin-eosin stained tissue sections from several organs. The mice were administered with GSK269962A at 10 mg/kg intraperitoneally for daily for 10 days. The organs (kidney, liver, lung and spleen) were fixed in formalin and assessed tissue morphology with hematoxylin and eosin (H&E) staining.

Table S1. AML and non-AML cell lines were treated with GSK269962A for 72 h. The IC_{50} values were determined by the CCK-8 assay.

**Cell line**	**IC_{50} ± SD (nM)**
MV4-11	0.61 ± 0.40
OCI-AML3	25.85 ± 1.55
Kasumi-1	170.13 ± 24.31
THP-1	427.77 ± 31.70
MOLM-13	705.90 ± 48.45
NOMO-1	726.93 ± 220.13
KG-1	1337.33 ± 192.14
U87	>10,000
SK-Hep1	>10,000

Table S2. Complete blood count (CBC) values.

	Control	GSK269962A
WBC (10^3/mm^3)	7.03 ± 2.11	5.59 ± 3.36
RBC (10^6/mm^3)	9.20 ± 0.79	8.80 ± 2.56
Hgb (g/dL)	13.82 ± 0.91	13.06 ± 3.82
HCT (vol%)	46.08 ± 3.50	44.50 ± 12.94
MCV (mg/dL)	50.14 ± 2.02	50.60 ± 2.04
MCH (pg)	15.02 ± 0.59	14.84 ± 0.64
MCHC (g/dL)	29.98 ± 0.48	29.32 ± 0.75
